# Highly Efficient Photocatalytic Z-Scheme Hydrogen Production over Oxygen-Deficient WO_3–x_ Nanorods supported Zn_0.3_Cd_0.7_S Heterostructure

**DOI:** 10.1038/s41598-017-06808-6

**Published:** 2017-07-26

**Authors:** Ammar Bin Yousaf, M. Imran, Syed Javaid Zaidi, Peter Kasak

**Affiliations:** 1 Center for Advanced Materials, Qatar University, Doha, 2713 Qatar; 2 Hefei National Laboratory for Physical Sciences at Microscale, University of Science and Technology of China, Hefei, Anhui 230026 PR China

## Abstract

The demand for clean renewable energy is increasing due to depleting fossil fuels and environmental concerns. Photocatalytic hydrogen production through water splitting is one such promising route to meet global energy demands with carbon free technology. Alternative photocatalysts avoiding noble metals are highly demanded. Herein, we fabricated heterostructure consist of oxygen-deficient WO_3–x_ nanorods with Zn_0.3_Cd_0.7_S nanoparticles for an efficient Z-Scheme photocatalytic system. Our as obtained heterostructure showed photocatalytic H_2_ evolution rate of 352.1 μmol h^−1^ with apparent quantum efficiency (AQY) of 7.3% at λ = 420 nm. The photocatalytic hydrogen production reaches up to 1746.8 μmol after 5 hours process in repeatable manner. The UV-Visible diffuse reflectance spectra show strong absorption in the visible region which greatly favors the photocatalytic performance. Moreover, the efficient charge separation suggested by electrochemical impedance spectroscopy and photocurrent response curves exhibit enhancement in H_2_ evolution rate. The strong interface contact between WO_3–x_ nanorods and Zn_0.3_Cd_0.7_S nanoparticles ascertained from HRTEM images also play an important role for the emigration of electron. Our findings provide possibilities for the design and development of new Z-scheme photocatalysts for highly efficient hydrogen production.

## Introduction

The increasing demand for energy and depleting crude oil resources forced researchers to find alternate options for rapidly growing world population. The burning of fossil fuels also deteriorating world’s climate by the emission of CO_2_ and other green house gases^1^. Therefore, a sustainable and clean energy source is the biggest challenge for the 21^st^ century. Photocatalytic hydrogen production emerges as environment friendly method since the pioneer work of Fujishima and Honda in 1972^2^. Since then, a large number of photocatalysts have been synthesized for water splitting to generate hydrogen^3^. However, most of catalysts either depend upon expensive noble metals as co-catalyst (Pt, Ru, and Rh) or only absorb in the ultraviolet region which accounts for only 4% of the incoming solar light. Metal oxides such as WO_3_, NiO and RuO_2_ emerges as new class of photocatalyst for efficient hydrogen production^4–9^. However, photocatalysts with maximum absorption in the visible region and suitable band-gap are highly desirable.

Cadmium sulphide (CdS) attracts considerable attention due to its narrow band gap (2.4 eV) for photocatalytic hydrogen evolution reaction. However the rate of H_2_ production over CdS is very low because of its photo-corrosion property and fast-recombination of electron–hole pair which renders its practical applications impossible^10^. The use of co-catalyst or incorporation of Zn ion into CdS to form Zn_1–x_Cd_x_S (0–1) greatly enhances the photocatalytic activity. Recently, the band-gap for Zn_1–*x*_Cd_*x*_S has been varied to achieve maximum visible absorption and greater charge separation efficiency for water splitting^11^. However, there are only few reports which suggest room temperature synthesis with excellent photocatalytic property and recyclability. Moreover, a heterostructure comprising two different photocatalysts is considered a better option compared to conventional system. The Z-scheme multi-component photocatalyst system was first introduced by Bard *et al*. in 1979 based on the concept of artificial photosynthesis^12^. In multi-component Z-scheme photocatalytic system, the photogenerated electrons from Photosystem I (PSI) in the conduction band transfer through the interface contact and recombine with the photogenerated holes in the valance band of Photosystem II (PSII). This system allows the visible light to use more efficiently and dramatically improves the photocatalytic H_2_ production^13^.

The photocatalytic reactions are surface bound reaction, in which photogenerated electron and holes takes part in the reaction. It is well known that oxygen vacancies in a non-stoichiometric crystal also carries two electrons each which can act as double electron donor^14^. There are few recent studies in which the role of surface oxygen vacancies (SOV) was investigated thoroughly for photo-catalytic water splitting^15^. Oxygen vacancies significantly impact the electronic properties of the material and shifts absorption towards longer wavelength. Oxygen vacancies also influences magnetic^16^, photocatalytic^17^, optical^18^, wettability^19^, and electrical^20^, properties of the catalyst. In a recent study, it is discovered that the oxygen vacancies improves the overall conductivity and enhances the adsorption of reactants on its surface for organic conversions^21^. Additionally, oxygen deficient metal oxide greatly enhances the visible-light driven hydrogen production by trapping the photo-induced charges and preventing the electron-hole recombination^22^. Mao and co-workers also created surface disorder in TiO_2_ by hydrogenation and increased solar absorption while promoting photocatalytic activity^23^. In other recent reports, oxygen-vacancies in TiO_2_, WO_3_, ZnO, and Fe_2_O_3_ also greatly enhance photocatalytic activity^24–27^.

Herein, we propose to fabricate a Z-scheme heterostructure consisting of oxygen deficient WO_3–x_ and Zn_0.3_Cd_0.7_S. Introduced oxygen vacancies in WO_3_ to form WO_3–x_/Zn_0.3_Cd_0.7_S heterostructure significantly increases the photocatalytic hydrogen production compare to heterostructure analogue without oxygen vacancies WO_3_/Zn_0.3_Cd_0.7_S. The rate of H_2_ evolution for WO_3–x_/Zn_0.3_Cd_0.7_S heterostructure is as high as 352.1 μmol h^−1^ with apparent quantum efficiency (AQY) of 7.3% at λ = 420 nm in repeatable manner from aqueous solution containing Na_2_SO_3_ and Na_2_S as sacrificial reagents.

## Results and Discussion

**Figure 1:**
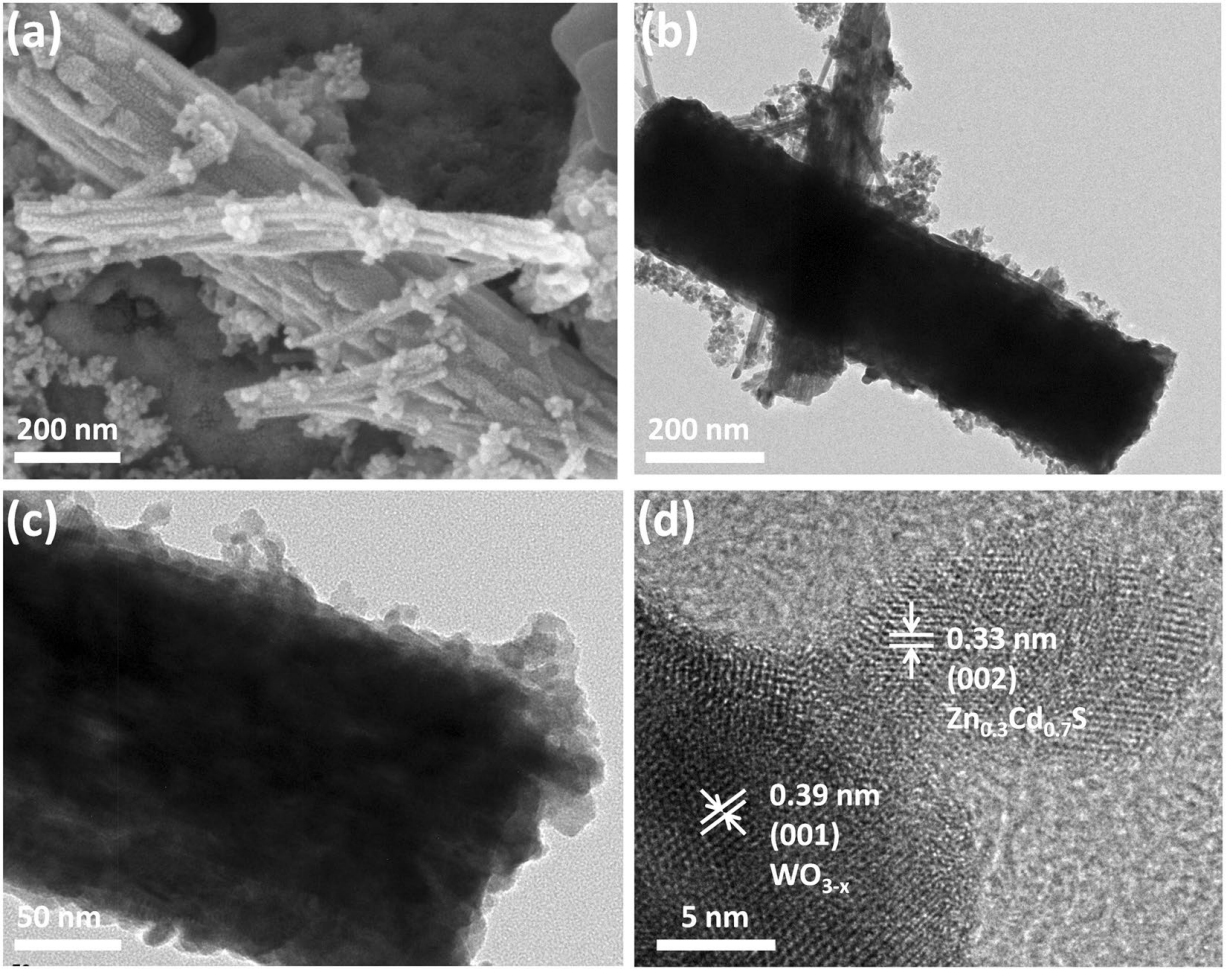
SEM image (**a**), TEM images (**b**,**c**), HRTEM images (**d**) of WO_3–x_/Zn_0.3_Cd_0.7_S heterostructure.

The morphology, size and structure of as synthesized WO_3–x_, Zn_0.3_Cd_0.7_S and WO_3–x_/Zn_0.3_Cd_0.7_S heterostructure were analyzed by scanning electron microscopy (SEM) and transmission electron microscopy (TEM) (Fig. 1, Figs S1 and S2). As shown in Fig. S1, the Zn_0.3_Cd_0.7_S are irregular shaped nanoparticles with a size of about 10–20 nm while the WO_3–x_ exhibit the shape of nanorods with a size in microns (Fig. S2). The SEM and TEM analysis of WO_3–x_/Zn_0.3_Cd_0.7_S heterostructure shows that Zn_0.3_Cd_0.7_S nanoparticles successfully anchored on the surface of WO_3–x_ nanorods (Fig. 1). The intimate contact between WO_3–x_ and Zn_0.3_Cd_0.7_S was observed by high resolution transmission electron microscopy (HRTEM) clearly demonstrating that WO_3–x_/Zn_0.3_Cd_0.7_S heterostructure with strong interface contact were successfully fabricated. The lattice spacing of 0.33 nm and 0.39 nm corresponds to (002) and (001) planes of Zn_0.3_Cd_0.7_S and WO_3–x_, respectively, which confirms the existence of heterostructure.

**Figure 2:**
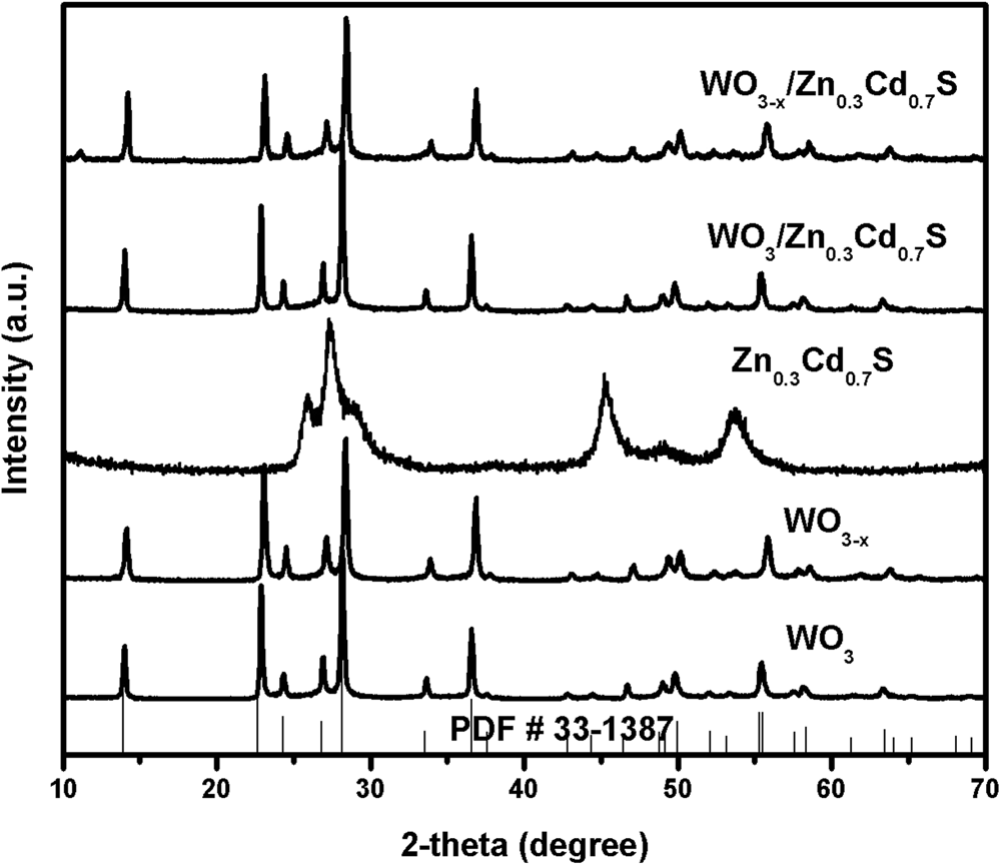
XRD pattern of as synthesized WO_3_, WO_3–x_, Zn_0.3_Cd_0.7_S, WO_3_/Zn_0.3_Cd_0.7_S and WO_3–x_/Zn_0.3_Cd_0.7_S heterostructure.

The XRD patterns of as synthesized samples are shown in Fig. 2. The XRD pattern of WO_3_ shows diffraction peaks which can be readily indexed to hexagonal Tungsten Oxide (JCPDS #33-1387) having cell parameters a = 7.298 Å, b = 7.298 Å, c = 3.899 Å and space group *P6/mmm* ^28^. The oxygen-deficient WO_3–x_ nanorods does not shows any clear difference in XRD pattern due to large size. The characteristic XRD diffraction peaks of Cd_0.3_Zn_0.7_S was easily observed at 28.1°, 45.7°, and 53.9° well matched with the (111), (220) and (311) planes of Zinc blend phase respectively. However, the characteristic diffraction peak of WO_3_ and WO_3–x_ in WO_3_/Zn_0.3_Cd_0.7_S and WO_3–x_/Zn_0.3_Cd_0.7_S were not observed may be due to low content, small size of Zn_0.3_Cd_0.7_S nanoparticles in the samples^29^.

**Figure 3:**
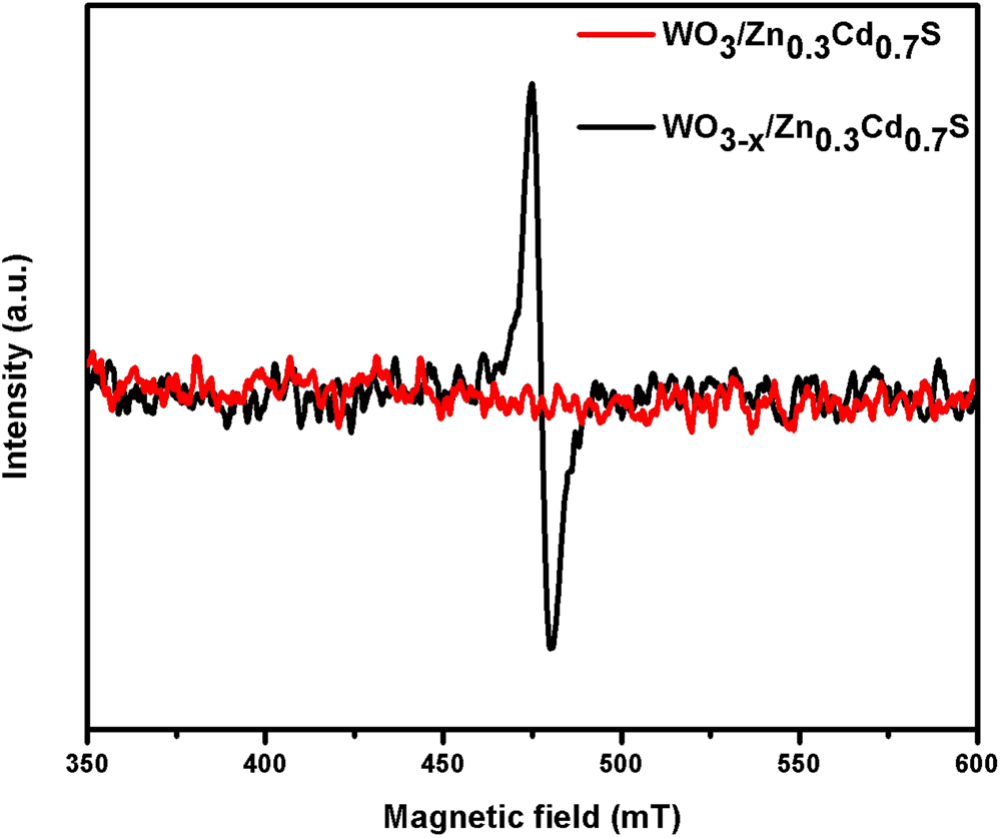
EPR spectra of the as synthesized WO_3_/Zn_0.3_Cd_0.7_S and WO_3–x_/Zn_0.3_Cd_0.7_S heterostructure recorded at T = 130 K.

Electron paramagnetic resonance (EPR) spectra of WO_3_/Zn_0.3_Cd_0.7_S and WO_3–x_/Zn_0.3_Cd_0.7_S were recorded to examine the paramagnetic character. (Fig. 3) The WO_3_/Zn_0.3_Cd_0.7_S sample does not shows any signal in EPR while WO_3–x_/Zn_0.3_Cd_0.7_S exhibits a sharp signal at g = 2.00 which can be readily assigned as electrons trapped on oxygen vacancies, thus generating an EPR signal at g = 2.00^30^.

**Figure 4:**
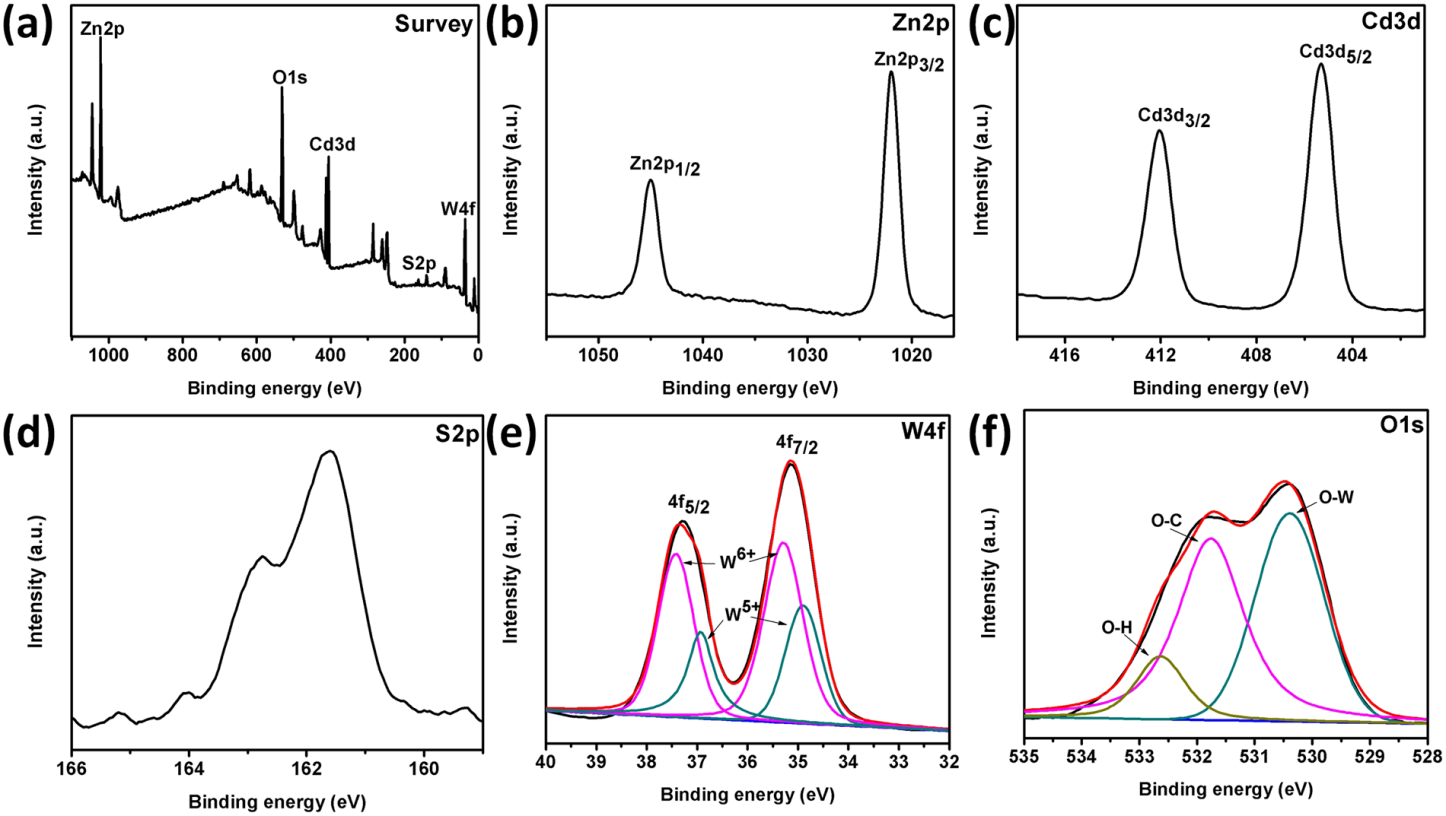
XPS spectra of WO_3–x_/Zn_0.3_Cd_0.7_S heterostructure, Survey spectrum, (**a**), Zn2p orbital (**b**), Cd3d orbital (**c**), S2p orbital (**d**), W4f orbital (**d**) and O1s orbital (**d**).

The Zn_0.3_Cd_0.7_S and WO_3–x_/Zn_0.3_Cd_0.7_S were also characterized by X-ray photoelectron spectroscopy (XPS) analysis as show in Figs 4 and S3. XPS survey spectrum of Zn_0.3_Cd_0.7_S shown in Fig. S3 indicate the presence of Zn, Cd and S in the nanoparticles. Figure S3b shows the XPS spectrum of Zn2p with the characteristic peak at 1023.6 and 1046.8 eV for Zn2p_3/2_ and Zn2p_1/2_, respectively. The high resolution XPS spectrum of Cd3d exhibit peaks at binding energy 405.1 and 411.8 eV corresponds to Cd3d_5/2_ and 3d_3/2_, respectively. The S2p orbital shows a broad peak at binding energy 161.3 eV which corresponding to S^2−^ ^31^. The ratio of Zn:Cd obtained from XPS analysis is well in agreement with the experimental calculations.

A typical survey XPS spectrum of WO_3–x_/Zn_0.3_Cd_0.7_S is also shown in Fig. 4a which confirms the coexistence of Zn, Cd, S, W, and O elements. The XPS spectra of Zn2p and Cd3d are plotted in Fig. 4b and c, respectively. The binding energies of Zn2p_3/2_ and Zn2p_1/2_ observed at 1021.9 and 1045.1 eV and Cd3d exhibits peaks at 405.2 eV and 412.1 eV corresponding to Cd3d_5/2_ and Cd3d_3/2_ respectively which agree well with the values reported for the divalent zinc and cadmium in pure metal sulphides. The S2p also show a peak at 161.5 eV which corresponds to S^2−^ in the sample^31^. (Fig. 4d) There is a difference in binding energies of Zn, Cd and S for pure Cd_0.3_Zn_0.7_S and WO_3–x_/Zn_0.3_Cd_0.7_S heterostructure, which suggest the difference in valence electron density and relaxation energy after heterostructure formation. The high resolution XPS spectrum of W4f orbital shows two strong peaks corresponding to W4f_7/2_ and W4f_5/2_. (Fig. 4e) The peaks can be further deconvoluted into two Gaussian components. The lower binding energy components centered at 34.8 eV and 36.9 eV represent W^5+^ while higher binding energy components at 35.3 eV and 37.4 eV matched with high oxidation state of tungsten (W^6+^)^32^. The presence of W^5+^ in XPS analysis is also consistent with the EPR data showing the existence of oxygen vacancies in the sample. Figure 4f shows O1s spectrum which can be deconvoluted into three Gaussian curves centered at 530.4 eV, 531.7 eV and 532.7 eV. The low binding energy component is ascribed to the O^2−^ ions in WO_3–x_ while the components at the higher binding energy region are related to the O-C from sample holder and physisorbed water molecules^33^.

**Figure 5:**
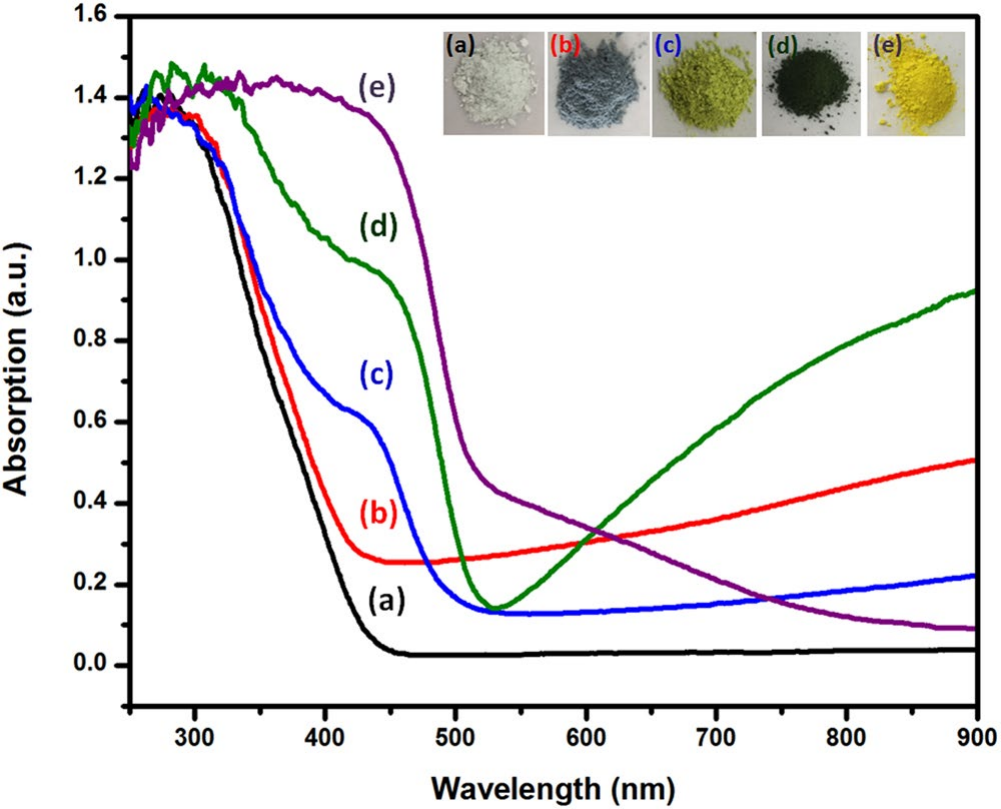
UV–visible diffuse reflectance spectra and inset photographs of WO_3_ (**a**), WO_3–x_ (**b**), Zn_0.3_Cd_0.7_S (**c**), WO_3_/Zn_0.3_Cd_0.7_S (**d**) and WO_3–x_/Zn_0.3_Cd_0.7_S heterostructure (**e**).

Figure 5 shows UV-vis diffuse reflectance spectra of WO_3_, WO_3–x_, Zn_0.3_Cd_0.7_S and WO_3_/Zn_0.3_Cd_0.7_S and WO_3–x_/Zn_0.3_Cd_0.7_S heterostructure. It can be clearly seen that WO_3_ shows a white color and almost no absorption in visible region while WO_3–x_ exhibit violet color and wide absorption band extending from 440 nm to 800 nm.

Additionally, Zn_0.3_Cd_0.7_S nanoparticles can absorb at wavelengths of about 475 nm in visible spectra which corresponds to a band gap of 2.61 eV. WO_3_/Zn_0.3_Cd_0.7_S displays a light green color, suggesting that the loading of Zn_0.3_Cd_0.7_S nanoparticles onto WO_3_ nanorods with combination with oxygen deficiency of WO_3–x_ increases the UV–visible absorption. It can be observed that oxygen deficient WO_3–x_/Zn_0.3_Cd_0.7_S heterostructure shows a deep brown color and strong absorption in the visible region which is beneficial for the photocatalytic performance. The WO_3–x_/Zn_0.3_Cd_0.7_S sample shows a larger red shift compared to WO_3_/Zn_0.3_Cd_0.7_S, owing to additional oxygen vacancies, which is also in agreement with EPR data.

**Figure 6:**
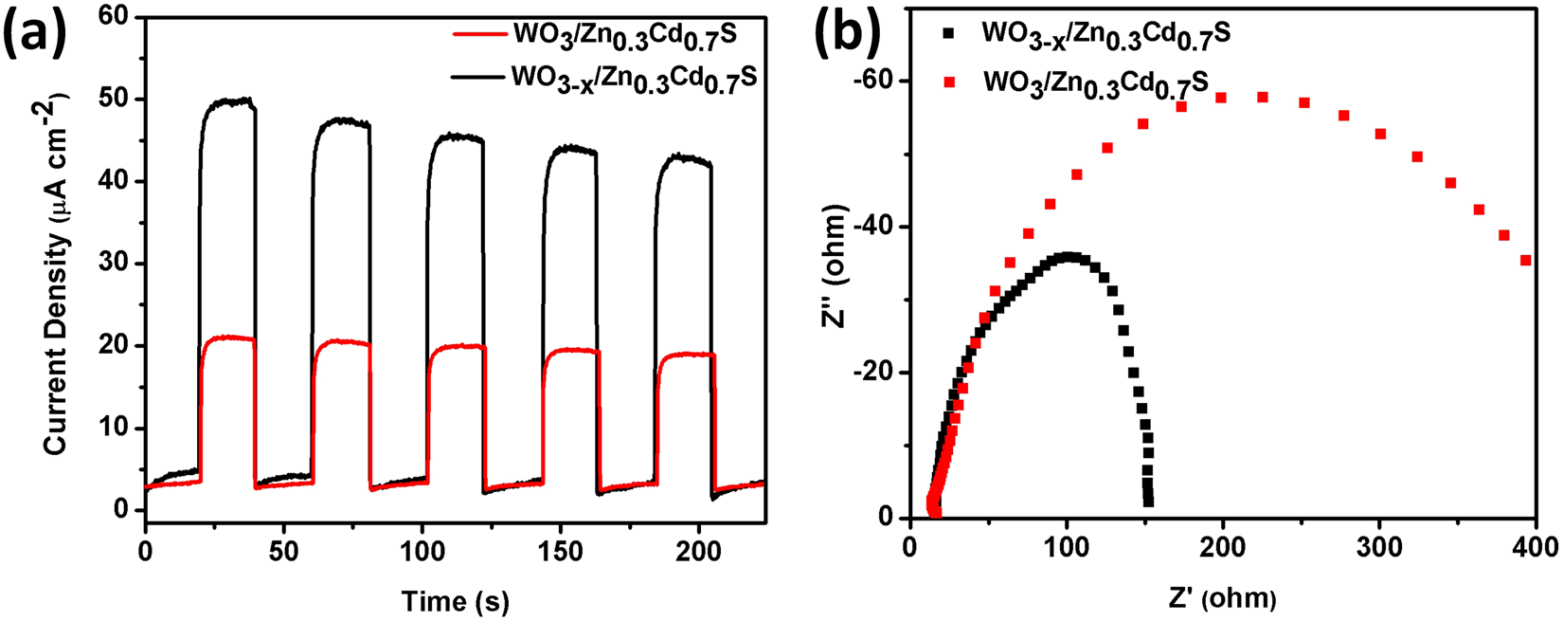
Photocurrent response vs. time and Nyquist plots for WO_3_/Zn_0.3_Cd_0.7_S and WO_3–x_/Zn_0.3_Cd_0.7_S heterostructure.

In addition, Transient photocurrent response curves of WO_3_/Zn_0.3_Cd_0.7_S and WO_3–x_/Zn_0.3_Cd_0.7_S were also examinated by photoelectrochemical test device under visible range for light with irradiation (λ ≥ 420 nm) using Ti foil. (see the Experimental section). As shown in Fig. 6a, the photocurrent value increases rapidly at a maximum value when the light turned on due to the separation of electron hole pairs at the heterostructure–electrolyte interface. The current value drops to zero when the light turned off, which is due to the recombination of electron-holes pairs. Notably, the WO_3–x_/Zn_0.3_Cd_0.7_S heterostructure shows much higher photocurrent than WO_3_/Zn_0.3_Cd_0.7_S, suggesting that the oxygen-deficient WO_3–x_ facilitate the charge transfer process resulting in higher photocatalytic performance^34^.

Electrochemical impedance spectroscopy (EIS) was also performed on a photoelectrochemical setup to investigate the charge separation efficiency of WO_3_/Zn_0.3_Cd_0.7_S and WO_3–x_/Zn_0.3_Cd_0.7_S heterostructure as is depicted in Fig. 6b. The large semicircle in the Nyquist plots represents the charge transfer process, while the smaller arc demonstrate fast separation of photogenerated electron–hole pairs and efficient interface charge flow^35^. As shown in Fig. 6b, the WO_3_/Zn_0.3_Cd_0.7_S have semicircle at the high frequency while WO_3–x_/Zn_0.3_Cd_0.7_S showed low charge transfer resistance (*Rct*) and much smaller semicircle suggesting the oxygen vacancies increase electrical conductivity and enhance visible-light driven hydrogen production^36, 37^. The oxygen vacancies thus providing a key role of rapid electron transfer for our Z-scheme photocatalytic H_2_ system. The results are also consistent with photocurrent response and diffuse UV-visible reflectance data.

**Figure 7:**
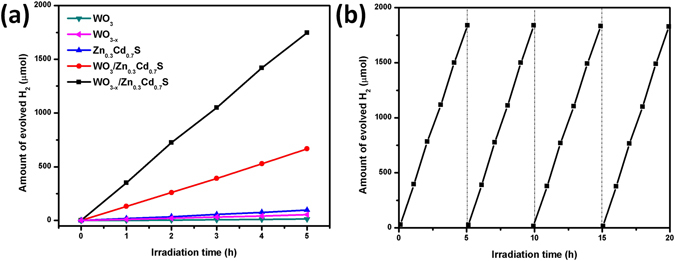
(**a**) Time courses of photocatalytic H_2_ evolution from Na_2_SO_3_, Na_2_S aq. solution on WO_3_, WO_3–x_, Zn_0.3_Cd_0.7_S, WO_3_/Zn_0.3_Cd_0.7_S and WO_3–x_/Zn_0.3_Cd_0.7_S heterostructure, (**b**) Time-cycle photocatalytic hydrogen production over WO_3–x_/Zn_0.3_Cd_0.7_S heterostructure under visible–light illumination (λ ≥ 420 nm).

The photocatalytic H_2_-evolution reactions of WO_3_, WO_3–x_, WO_3_/Zn_0.3_Cd_0.7_S and WO_3–x_/Zn_0.3_Cd_0.7_S were performed in the presence of Na_2_S and Na_2_SO_3_ as sacrificial reagents under xenon lamp irradiation (Figure 7a). WO_3_ nanorods exhibit very low H_2_-evolution rate (2 μmol h^−1^). The band edge position of γ-monoclinic phase of WO_3_ is not optimal for photocatalytic water splitting, however, the band edge of hexagonal-WO_3_ match up with the redox potential of water, which is used in our case and exhibit very low H_2_-evolution rate while the oxygen deficient WO_3–x_ shows a better performance for H_2_ production (10 μmol h^−1^) implying the presence of oxygen vacancies leads to increase in photocatalytic activity^38^. Zn_0.3_Cd_0.7_S nanoparticles alone showed moderate photocatalytic H_2_-evolution rate (19.2 μmol h^−1^). Remarkably, the WO_3_/Zn_0.3_Cd_0.7_S Z-scheme system showed an improvement in hydrogen production under visible light with H_2_-evolution rate of 132 μmol h^−1^. The defect rich WO_3–x_/Zn_0.3_Cd_0.7_S heterostructure demonstrates highest rate of H_2_ production 352.1 μmol h^−1^ with apparent quantum efficiency (AQY) of 7.3% at 420 nm. The photocatalytic hydrogen production reaches up to 1746.8 μmol in 5 h process which is considerably higher than some previous reports on photocatalytic H_2_ production such as TiO_2_/Pt/P_2_W_17_^39^, TiO_2_/Au/CdS^40^, ZnO/Au/CdS^41^, WO_3_/PbBi_2_Nb_1.9_Ti_0.1_O_9_^42^, BiVO_4_/Ru-SrTiO_3_^43^ which showed rate 19.6, 3.2, 60.8, 14.8 and 22 μmol h^−1^, respectively. It is worth mentioning that no noble metals were used in our system^44–46^. Moreover, as it is seen from Fig. 7b, the process for production is repeatable and after 4 cycles was not observed decreasing in activities confirming stability, robustness and durability of this systems. The UV-vis diffuse reflectance spectra of oxygen deficient WO_3–x_ nanorods show a prominent color change and strong absorption in the visible region which is favorable for the photocatalytic hydrogen production. The photocurrent response and EIS results also demonstrate more efficient photoelectron emigration in oxygen deficient-WO_3–x_/Zn_0.3_Cd_0.7_S heterostructure compared to oxygen-vacancy-free WO_3_/Zn_0.3_Cd_0.7_S sample. The fast electron transfer efficiently captures the photo induced holes in the valence band (VB) of Zn_0.3_Cd_0.7_S. The fast recombination of e/h by the photo-generated electrons from conduction band (CB) of WO_3–x_ and holes from the VB of Zn_0.3_Cd_0.7_S results in excellent photocatalytic performance. For our oxygen-deficient WO_3–x_/Zn_0.3_Cd_0.7_S Z-scheme system, the photo-generated holes tend to be present in the VB WO_3–x_, while the electrons in the conduction band of WO_3–x_ combine with the holes of Zn_0.3_Cd_0.7_S through the interface contact. As a result, the photo-generated charge carrier recombination can be significantly decreased. Therefore, more electrons in the CB of Zn_0.3_Cd_0.7_S are available to reduce H^+^ to H_2_, which results in highly efficient H_2_ production^47^. The visible light absorption can be attributed to the electron transition from the WO_3–x_ valence band to the new oxygen vacancy energy bands near Fermi level, consequently, the electronic band gap decreases. Therefore, it is possible to use less energy per time to activate the photoelectrons and thus effectively improve the carrier separation efficiency^48, 49^. Oxygen vacancies also carries two electrons each which can act as double electron donors to capture photo-generated holes and enhances the H_2_ evolution performance. The strong interface contact in WO_3–x_/Zn_0.3_Cd_0.7_S ascertained from HRTEM images also strongly favors the efficient charge movement. We have also tested our as synthesized WO_3–x_/Zn_0.3_Cd_0.7_S heterostructure for photocatalytic O_2_ production which shows 3.24 μmol h^−1^ (Figure S4). However, the photocatalytic O_2_ production is not as high as H_2_ production.

**Figure 8:**
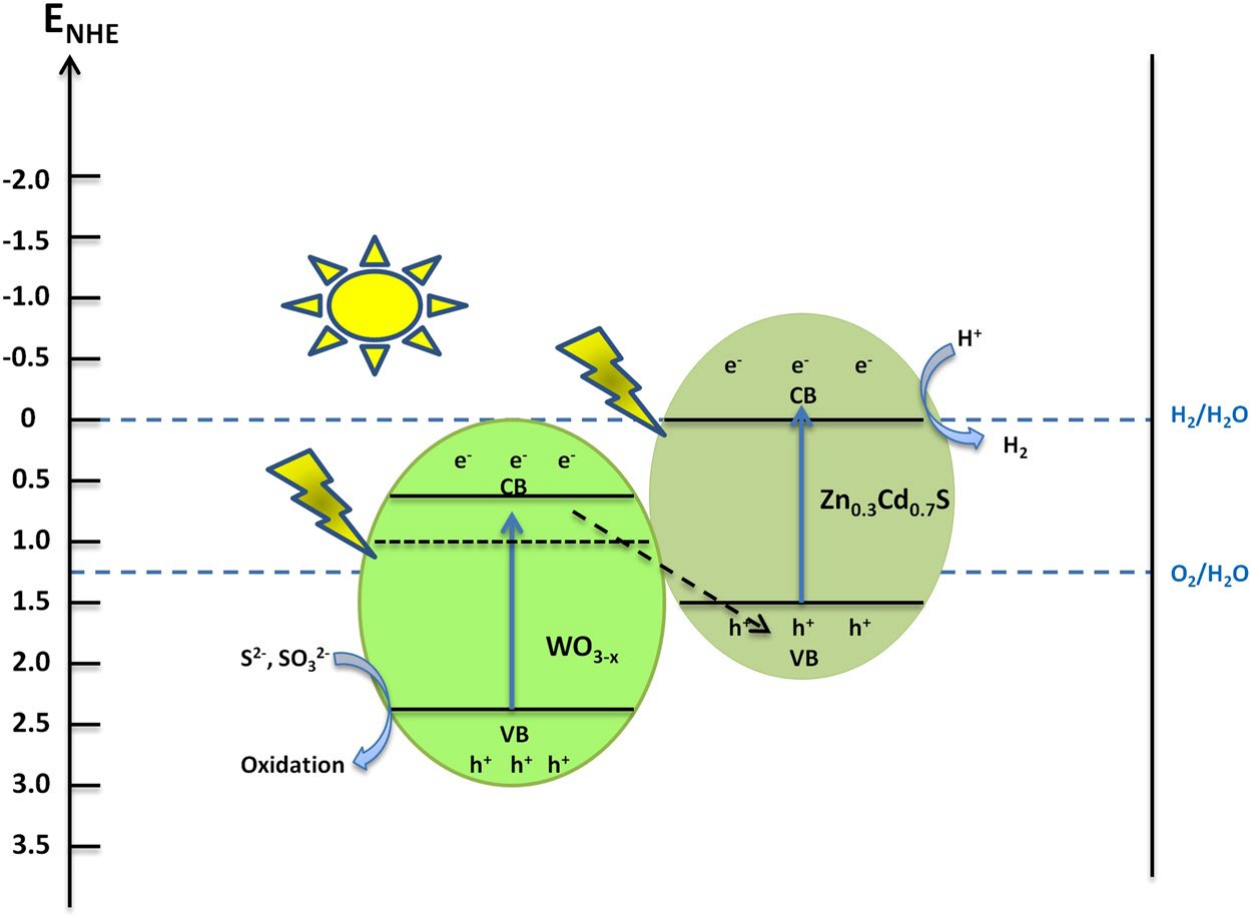
A schematic presentation of the charge transfer process for WO_3–x_/Zn_0.3_Cd_0.7_S heterostructure.

The recyclability of any materials is an important marker to which it is applicable for practical applications. It has been observed that metal sulphides usually exhibit photo-corrosion property when used for prolonged photocatalytic hydrogen production. In order to analyze the stability of our as-synthesized catalyst, we also performed time course of photocatalytic hydrogen production under similar conditions. As was mentioned previously, our Z-scheme WO_3–x_/Zn_0.3_Cd_0.7_S heterostructure shows excellent stability for H_2_ evolution. The photo-generated electrons from WO_3–x_ and oxygen vacancies also suppress the oxidation of Zn_0.3_Cd_0.7_S, thus inhibiting the photo-corrosion property resulting in enhanced photocatalytic performance. Previous reports showed that when there are small amount of oxygen vacancies in oxygen deficient material, the defect energy level is usually below the conduction band^50^. However in our case the, the concentration of oxygen vacancies in WO_3–x_ is much higher (x = 0.4) thus the defect energy level delocalized over the conduction band. The Z-scheme electron transfer pathway needs to satisfy three conditions; i.e. PS I can only produce O_2_, PS II can only produce H_2_ and overall water splitting can occur in the presence of PS I and PS II. WO_3_ have been reported to produce O_2_ while Zn_1-x_Cd_x_S have shown potential to be used for photocatalytic H_2_ production^51, 52^. We have also tested photocatalytic O_2_ production using WO_3–x_/Zn_0.3_Cd_0.7_S heterostructure which shows 3.24 μmol h^−1^ and reaches up to 15.45 μmol after 5 hours. Moreover, the defect rich WO_3–x_/Zn_0.3_Cd_0.7_S heterostructure also demonstrates highest rate of H_2_ production 352.1 μmol h^−1^. This indicates that the photogenerated holes in the VB of WO_3–x_ and photogenerated electrons in the CB of Zn_0.3_Cd_0.7_S are used to oxidize and reduce water into O_2_ and H_2_, respectively. Meanwhile, the photogenerated electrons in the CB of WO_3–x_ recombine with the photogenerated holes in the VB of Zn_0.3_Cd_0.7_S through the solid-solid interface contact^53^. Meanwhile, the photo-generated electrons in the conduction band of Zn_0.3_Cd_0.7_S reduces H^+^ to H_2_ while the photo-generated holes in conduction band of WO_3–x_ trapped by sacrificial reagents which undergo the oxidation of SO_3_^2−^ to SO_4_^2−^ ^54^ (Figure 8). This possible Z-scheme mechanism is also consistent with the experimental data, EIS and transient photocurrent response results and various previous reports^55–59^. Our multi-component Z-scheme system provides advancement in the design and development of low-cost highly efficient photocatalytic materials for water splitting.

## Conclusions

In conclusion, we have synthesized a unique no noble metal Z-scheme photocatalyst by fabrication of oxygen-deficient WO_3–x_ nanorods with Zn_0.3_Cd_0.7_S nanoparticles. The WO_3–x_/Zn_0.3_Cd_0.7_S heterostructure shows visible light driven hydrogen production of 352.1 μmol h^−1^ with apparent quantum efficiency (AQY) of 7.3% at 420 nm. The photocatalytic H_2_ evolution reaches up to 1746.8 μmol after 5 hours in repeatable manner without decreasing activites over 4 cycles. The EIS and photocurrent response results suggest efficient charge separation which is key factor for the enhancement of the activity. The solid-solid interfacial contact between WO_3–x_ and Zn_0.3_Cd_0.7_S favors photo-generated electron emigration from the conduction band of WO_3–x_ to the valance band of Zn_0.3_Cd_0.7_S, thus capturing the photogenerated holes. The remaining photogenerated electrons in the conduction band of Zn_0.3_Cd_0.7_S efficiently reduce H^+^ to H_2_. Overall the hydrogen evolution rate over oxygen-deficient WO_3–x_/Zn_0.3_Cd_0.7_S heterostrcture is considerably high compared to WO_3_/Zn_0.3_Cd_0.7_S and without the use of noble metals. Our approach opened up new avenue for to synthesis Z-scheme photocatalytic system for efficient hydrogen production from water splitting.

## Experimental

### Chemicals

Sodium tungstate (Na_2_WO_4_), Sodium sulfate (Na_2_SO_4_), Zinc acetate (Zn(CH_3_COO)_2_ · 2H_2_O), Cadmium acetate (Cd(CH_3_COO)_2_ · 2H_2_O), Sodium sulfide nonahydrate (Na_2_S . 9H_2_O), Sodium sulphite (Na_2_SO_3_) were purchased from Sinopharm Chemical Reagent. All other chemical reagents were of analytical grade and used as received without further purification.

### Characterization

The morphology of the particles was observed by scanning electron microscope (SEM, JSM 6700F, JEOL). Transmission electron microscopic (TEM) images and high-resolution transmission electron microscopic (HRTEM) images were carried out on a JEM-2100F field emission electron microscope at an accelerating voltage of 200 kV. The X-ray powder diffraction (XRD) patterns of the products were performed on a Philips X’Pert Pro Super diffractometer with Cu-Kα radiation (λ = 1.54178 Å). The operation voltage was maintained at 40 kV and current at 200 mA, respectively. The X-ray photoelectron spectroscopy (XPS) was carried out on a PerkinElmer RBD upgraded PHI-5000C ESCA system. A Shimadzu spectrophotometer (Model 2501 PC) was used to record the UV–vis diffuse reflectance spectra of the samples in the region of 200 to 800 nm. The electron paramagnetic resonance (EPR) spectra were recorded on a JEOL JES-FA200 EPR spectrometer (140 K, 9064 MHz, 0.998 mW, X-band).

### Synthesis

Heterostructure WO_3–x_/Zn_0.3_Cd_0.7_S was synthesized in simple three steps. In the first hexagonal WO_3_ nanorods were prepared by hydrothermal reaction. Subsequently calcination in Ar/H_2_ environment was carried out to results WO_3–x_. Finally Zn_0.3_Cd_0.7_S was introduced to nanorod surface by reaction of Zn^2+^, Cd^2+^ precursors and Na_2_S in alkali conditions. Detailed synthesis is as follows:

### Synthesis of WO_3–x_ Nanorods

WO_3–x_ nanorods were synthesized by adding 0.1 g of Sodium tungstate and 0.05 g of Sodium sulfate in 4 ml of water followed by drop wise addition of 0.5 M HCl to adjust the pH value of the solution to 2.0. Then, the solution was poured into Teflon-lined stainless steel autoclave and heated 190 °C for 24 h. After cooling down the autoclave, the products was obtained by centrifugation and washed thoroughly with water, ethanol and dried at 60 °C. The centrifuge material was further treated for calcinations in a furnace for 2 h at 350 °C in Ar/H_2_ environment (10 mL min^−1^) with heating rate of 5 °C min^−1^. The final product obtained was further used for characterizations, heterostructure synthesis and applications. The synthesis of WO_3_ nanorods was same except the calcinations process.

### Synthesis of WO_3–x_/Zn_0.3_Cd_0.7_S Heterostructure

In a typical synthesis, 50 mg WO_3–x_ nanorods were dispersed in 100 mL of distilled water, then a certain amount of Zn(CH_3_COO)_2_ · 2H_2_O and Cd(CH_3_COO)_2_ · 2H_2_O were added and pH of the solution was adjusted to 7.4 using 0.1 M sodium hydroxide. After 10–15 minutes, aqueous solution of sodium sulfide (Na_2_S . 9H_2_O) was drop wise added into above solution. The resultant mixture was stirred at room temperature for 24 h. The obtained powders were washed with water and ethanol and dried in oven at 60 °C. The Synthesis of WO_3_/Zn_0.3_Cd_0.7_S heterostructure was same and bare Zn_0.3_Cd_0.7_S nanoparticles were also prepared following the same procedure except the use of WO_3–x_. The optimum ratio of WO_3_, WO_3–x_ and Zn_0.3_Cd_0.7_S in WO_3_/Zn_0.3_Cd_0.7_S, and WO_3–x_/Zn_0.3_Cd_0.7_S heterostructure was obtained to be 1:1.2 and 1:1.2, respectively.

### Photocatalytic reaction

The photocatalytic H_2_ evolution from water splitting was performed in a vacuumed, gas–closed circulation system using 300 W Xe lamp equipped with a λ ≥ 420 nm cut–off filter. The average light intensity was 2.84 mW/cm^2^. In a typical procedure, 100 mg of catalyst was dispersed in 100 mL water containing 0.1 M Na_2_S and 0.1 M Na_2_SO_3_. The on–line gas chromatography (Agilent, 6820, TCD detector, N_2_ carrier) was used to determine the amount of hydrogen evolved and compared with other samples. The photocatalytic O_2_ production performed vacuumed, gas–closed circulation system in 5 mM KIO_3_ solution using 300 W Xe lamp equipped with a λ ≥ 420 nm cut–off filter. The on–line gas chromatography (Agilent, 6820, TCD detector, N_2_ carrier) was used to determine the amount of oxygen evolved.

### Quantum efficiency measurement

Apparent quantum yields (AQYs) were determined using a 420 nm band pass filter. The number of incident photons from the Xenon lamp were measured with a power meter (1831-R, Newport). Apparent quantum yields (AQYs) were calculated by the following equation:

$$AQY\,( \% )=\,\frac{2\times The\,number\,of\,evolved\,{H}_{2}\,molecules}{The\,number\,of\,incident\,photons}\,\times 100$$

### Electrochemical and Photo-electrochemical measurements

Electrochemical and photoelectrochemical measurements were conducted in 0.1 M Na_2_SO_4_ electrolyte solution with a three-electrode quartz cell. Ag/AgCl was used as reference electrode while platinum wire was used as counter electrode and catalysts film electrodes on Ti foil worked as working electrode. The catalysts films were prepared by dropping catalyst suspensions (10 mg mL^−1^ in ethanol) onto Ti foil by following doctor-blade coating method with a glass rod and scotch tape and resultant electrodes were annealed for 12 h at 90 °C. For the measurements, the electrodes were pressed against an electrochemical cell with a working area of 4.0 cm^2^. Photo-electrochemical test systems were composed of a CHI 660B electrochemistry potentiostat (Shanghai Chenhua Limited, China).

## Electronic supplementary material

Supplementary Information

## References

[1] Ni M, Leung MKH, Leung DYC, Sumathy K (2007). A Review and Recent Developments in Photocatalytic Water–Splitting using TiO_2_ for Hydrogen Production. Renew. Sust. Energ. Rev..

[2] Fujishima A, Honda K (1972). Electrochemical Photolysis of Water at a Semiconductor Electrode. Nature.

[3] Hoffmann MR, Martin ST, Choi W, Bahnemann DW (1995). Environmental Applications of Semiconductor Photocatalysis. Chem. Rev..

[4] Abe R, Sayama K, Arakawa H (2003). Significant Effect of Iodide Addition on Water Splitting into H_2_ and O_2_ over Pt–loaded TiO_2_ Photocatalyst: Suppression of Backward Reaction. Chem. Phys. Lett..

[5] Sato J (2005). RuO_2_-Loaded β- Ge_3_N_4_ as a Non-Oxide Photocatalyst for Overall Water Splitting. J. Am. Chem. Soc..

[6] Iwashina K, Kudo A (2011). Rh-Doped SrTiO_3_ Photocatalyst Electrode Showing Cathodic Photocurrent for Water Splitting under Visible–Light Irradiation. J. Am. Chem. Soc..

[7] Hwang DW, Kim J, Park TJ, Lee JS (2002). Mg–Doped WO_3_ as a Novel Photocatalyst for Visible Light–Induced Water Splitting. Catal. Lett..

[8] Kato H, Kudo A (2001). Water Splitting into H_2_ and O_2_ on Alkali Tantalate Photocatalysts ATaO_3_ (A = Li, Na, and K). J. Phys. Chem. B.

[9] Maeda K, Saito N, Lu DL, Inoue Y, Domen K (2007). Photocatalytic Properties of RuO_2_-Loaded β-Ge_3_N_4_ for Overall Water Splitting. J. Phys. Chem. C.

[10] Zong X (2008). Enhancement of Photocatalytic H_2_ Evolution on CdS by Loading MoS_2_ as Cocatalyst under Visible Light Irradiation. J. Am. Chem. Soc..

[11] Hsu YY (2015). Heterojunction of Zinc Blende/Wurtzite in Zn_1−x_Cd_x_S Solid Solution for Efficient Solar Hydrogen Generation: X−ray Absorption/Diffraction Approaches. ACS Appl. Mater. Interfaces.

[12] Allen JB (1979). Photoelectrochemistry and heterogeneous photo-catalysis at semiconductors. J. Photochem..

[13] Li HJ, Tu WG, Zhou Y, Zou ZG (2016). Z-Scheme Photocatalytic Systems for Promoting Photocatalytic Performance: Recent Progress and Future Challenges. Adv. Sci..

[14] Zou XX (2013). Facile Synthesis of Thermal and Photostable Titania with Paramagnetic Oxygen Vacancies for Visible-Light Photocatalysis. Chem. – Eur. J..

[15] Pan XY, Yang MQ, Fu XZ, Zhang N, Xu YJ (2013). Defective TiO_2_ with oxygen vacancies: synthesis, properties and photocatalytic applications. Nanoscale.

[16] Mal S, Nori S, Narayan J, Prater JT (2011). Defect-mediated ferromagnetism and controlled switching characteristics in ZnO. J. Mater. Res..

[17] Bayati MR, Ding J, Lee YF, Narayan RJ, Narayan J (2012). Defect mediated photocatalytic decomposition of 4-chlorophenol on epitaxial rutile thin films under visible and UV illuminations. J. Phys. C: Solid State Phys..

[18] Bayati MR, Joshi S, Narayan RJ, Narayan J (2013). Low temperature processing and control of structure and properties of epitaxial TiO_2_/Sapphire thin film heterostructures. J. Mater. Res..

[19] Bayati MR, Joshi S, Molaei R, Narayan RJ, Narayan J (2013). Ultrafast switching in wetting properties of TiO_2_/YSZ/Si (001) heteroepitaxy induced by laser irradiation. J. Appl. Phys..

[20] Gupta P, Dutta T, Mal S, Narayan J (2012). Controlled p-type to n-type conductivity transformation in NiO thin films by ultraviolet-laser irradiation. J. Appl. Phys..

[21] Yu HW (2017). Structure–activity relationships of Cu–ZrO_2_ catalysts for CO_2_ hydrogenation to methanol: interaction effects and reaction mechanism. RSC Adv..

[22] Vitaly G, Su CY, Perng TP (2015). Surface reconstruction, oxygen vacancy distribution and photocatalytic activity of hydrogenated titanium oxide thin film. J. Catal..

[23] Chen X, Liu L, Yu PY, Mao SS (2011). Increasing Solar Absorption for Photocatalysis with Black Hydrogenated Titanium Dioxide Nanocrystals. Science.

[24] Wang Z (2013). Visible-Light Photocatalytic, Solar Thermal, and Photoelectrochemical Properties of Aluminium-Reduced Black Titania. Energy Environ. Sci..

[25] Liu G (2013). Enhancement of Visible-Light-Driven O_2_ Evolution from Water Oxidation on WO_3_ Treated with Hydrogen. J. Catal..

[26] Lv Y (2013). The Surface Oxygen Vacancy Induced Visible Activity and Enhanced UV Activity of a ZnO_1−x_ Photocatalyst. Catal. Sci. Technol..

[27] Ling Y (2012). The Influence of Oxygen Content on the Thermal Activation of Hematite Nanowires. Angew. Chem., Int. Ed..

[28] Her YC, Chang CC (2014). Facile synthesis of one-dimensional crystalline/amorphous tungsten oxide core/shell heterostructures with balanced electrochromic properties. CrystEngComm..

[29] Akhtar MS, Malik MA, Riaz S, Naseem S (2015). Room temperature ferromagnetism and half metallicity in nickel doped ZnS: Experimental and DFT studies. Mater. Chem. Phys..

[30] Nakamura I (2000). Role of oxygen vacancy in the plasma-treated TiO_2_ photocatalyst with visible light activity for NO removal. J. Mol. Catal. A: Chem..

[31] Xu X (2012). Novel mesoporous Zn_x_Cd_1−x_S nanoparticles as highly efficient photocatalysts. Appl. Catal. B: Environ..

[32] Zhu J (2017). Intrinsic Defects and H Doping in WO_3_. Sci. Rep..

[33] Pavel VK (2015). XPS study of surface chemistry of tungsten carbides nanopowders produced through DC thermal plasma/hydrogen annealing process. Appl. Surf. Sci..

[34] Zhou X (2016). New Co(OH)_2_/CdS nanowires for efficient visible light photocatalytic hydrogen production. J. Mater. Chem. A.

[35] Ye RQ (2016). Fabrication of CoTiO_3_/g-C_3_N_4_ Hybrid Photocatalysts with Enhanced H_2_ Evolution: Z-Scheme Photocatalytic Mechanism Insight. ACS Appl. Mater. Interfaces.

[36] Wang Q (2016). Scalable water splitting on particulate photocatalyst sheets with a solar-to-hydrogen energy conversion efficiency exceeding 1%. Nat. Mater..

[37] Yang TH (2013). High Density Unaggregated Au Nanoparticles on ZnO Nanorod Arrays Function as Efficient and Recyclable Photocatalysts for Environmental Purification. Small.

[38] Lee TH, Lee YH, Jang WS, Aloysius S (2016). Understanding the advantage of hexagonal WO_3_ as an efficient photoanode for solar water splitting: a first-principles perspective. J. Mater. Chem. A.

[39] Ning FN, Jin ZJ, Wu YQ, Lu GQ, Li DY (2011). Z-Scheme Photocatalytic System Utilizing Separate Reaction Centers by Directional Movement of Electrons. J. Phys. Chem. C.

[40] Ding L (2013). Butterfly wing architecture assisted CdS/Au/TiO_2_ Z-scheme type photocatalytic water splitting. Int. J. Hydrogen Energ..

[41] Yu ZB (2013). Self-assembled CdS/Au/ZnO heterostructure induced by surface polar charges for efficient photocatalytic hydrogen evolution. J. Mater. Chem. A.

[42] Kim HG (2006). Photocatalytic ohmic layered nanocomposite for efficient utilization of visible photons. Appl. Phys. Lett..

[43] Sasaki Y, Nemoto H, Saito K, Kudo A (2009). Solar Water Splitting Using Powdered Photocatalysts Driven by Z-Schematic Interparticle Electron Transfer without an Electron Mediator. J. Phys. Chem. C.

[44] Liu C, Tang JY, Chen HM, Liu B, Yang PD (2013). A Fully Integrated Nanosystem of Semiconductor Nanowires for Direct Solar Water Splitting. Nano Lett..

[45] Hidehisa H, Takanori I, Shintaro I, Tatsumi I (2011). Long-time charge separation in porphyrin/KTa(Zr)O_3_ as watersplitting photocatalyst. Phys. Chem. Chem. Phys..

[46] Ma SSK (2013). A Redox-Mediator-Free Solar-Driven Z-Scheme Water-Splitting System Consisting of Modified Ta_3_N_5_ as an Oxygen-Evolution Photocatalyst. Chem. Eur. J..

[47] Yu JG, Wang SH, Low JX, Xiao W (2013). Enhanced photocatalytic performance of direct Z-scheme g-C_3_N_4_–TiO_2_ photocatalysts for the decomposition of formaldehyde in air. Phys. Chem. Chem. Phys..

[48] Dong LP, Jia RX, Xin B, Peng B, Zhang YM (2017). Effects of oxygen vacancies on the structural and optical properties of β-Ga_2_O_3_. Sci Rep..

[49] Gan JY (2013). Oxygen vacancies promoting photoelectrochemical performance of In_2_O_3_ nanocubes. Sci Rep..

[50] Zheng YH (2007). Luminescence and Photocatalytic Activity of ZnO Nanocrystals: Correlation between Structure and Property. Inorg. Chem..

[51] Wang YL (2017). Controllable Synthesis of Hexagonal WO_3_ Nanoplates for Efficient Visible-Light-Driven Photocatalytic Oxygen Production. Chem. Asian J..

[52] Li Q (2013). Zn_1–x_Cd_x_S Solid Solutions with Controlled Bandgap and Enhanced Visible-Light Photocatalytic H_2_-Production Activity. ACS Catal..

[53] Zhou P, Yu JG, Mietek J (2014). All-Solid-State Z-Scheme Photocatalytic Systems. Adv. Mater..

[54] Wang XW (2011). TiO_2_ films with oriented anatase {001} facets and their photoelectrochemical behavior as CdS nanoparticle sensitized photoanodes. J. Mater. Chem..

[55] Yu WL, Xu DF, Peng TY (2015). Enhanced photocatalytic activity of g-C_3_N_4_ for selective CO_2_ reduction to CH_3_OH via facile coupling of ZnO: a direct Z-scheme mechanism. J. Mater. Chem. A.

[56] Jin J, Yu JG, Gu DP, Cui C, Ho WK (2015). A Hierarchical Z‐Scheme CdS–WO_3_ Photocatalyst with Enhanced CO_2_ Reduction Activity. Small.

[57] Pan L (2017). Highly efficient Z-scheme WO_3–x_ quantum dots/TiO_2_ for photocatalytic hydrogen generation. Chin. J. Catal..

[58] Cui LF (2017). Facile preparation of Z-scheme WO_3_/g-C_3_N_4_ composite photocatalyst with enhanced photocatalytic performance under visible light. Appl. Surf. Sci..

[59] Liu JJ, Cheng B, Yu JG (2016). A new understanding of the photocatalytic mechanism of the direct Z-scheme g-C_3_N_4_/TiO_2_ heterostructure. Phys. Chem. Chem. Phys..

