# Supplementary material for: Highly Efficient Photocatalytic Z-Scheme Hydrogen Production over Oxygen-Deficient WO3–x Nanorods supported Zn0.3Cd0.7S Heterostructure
Source: Sci Rep. 2017 Jul 26;7:6574. doi: 10.1038/s41598-017-06808-6 (PMC5529397; doi:10.1038/s41598-017-06808-6)
Supplement: Supplementary file 1 — Supplementary Information [file 41598_2017_6808_MOESM1_ESM.doc]

Supporting information

Highly Efficient Photocatalytic Z-Scheme Hydrogen Production over Oxygen-Deficient WO_3-x_ Nanorods supported Zn_0.3_Cd_0.7_S Heterostructure

Ammar B. Yousaf^1,‡,*^, M. Imran,^2,‡^ Syed Javaid Zaidi ^1^ and Peter Kasak ^1,*^

^1^ Centre for Advanced Materials, Qatar University, Doha 2713, Qatar

^2^ Hefei National Laboratory for Physical Sciences at Microscale, University of Science and Technology of China, Hefei, Anhui 230026, PR China

^‡^These two authors contributed equally to this work.

Correspondence and requests for materials should be addressed to A. B. Yousaf & P. Kasak

*Email: [ammar@mail.ustc.edu.cn](mailto:ammar@mail.ustc.edu.cn) / [muhammad.ammar@qu.edu.qa](mailto:muhammad.ammar@qu.edu.qa) / [ammar.chemist18@gmail.com](mailto:ammar.chemist18@gmail.com) (A.B. Yousaf),

[peter.kasak@qu.edu.qa](mailto:peter.kasak@qu.edu.qa) (P. Kasak)


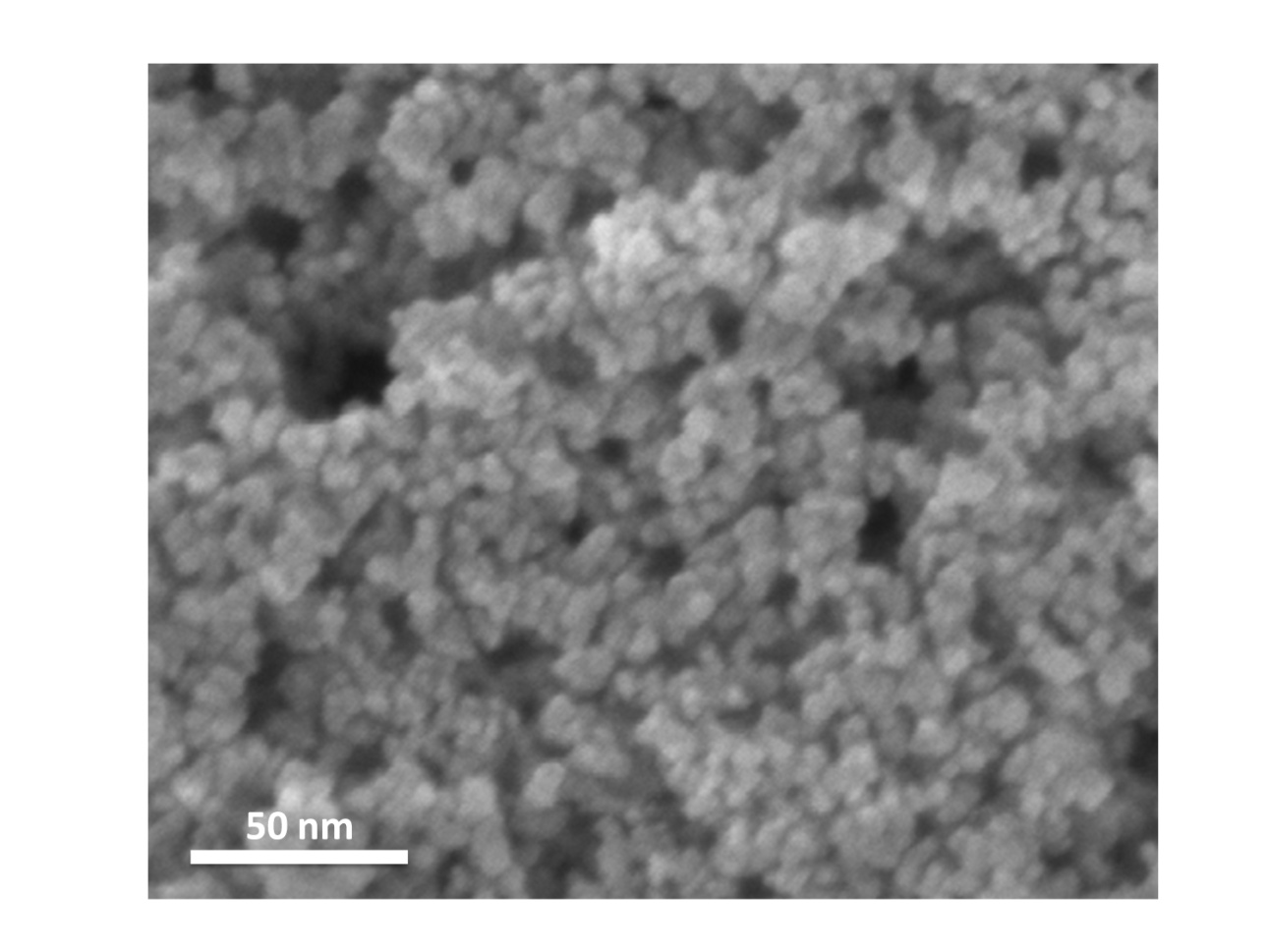


**Figure S1:** SEM image Zn_0.3_Cd_0.7_S nanoparticles.


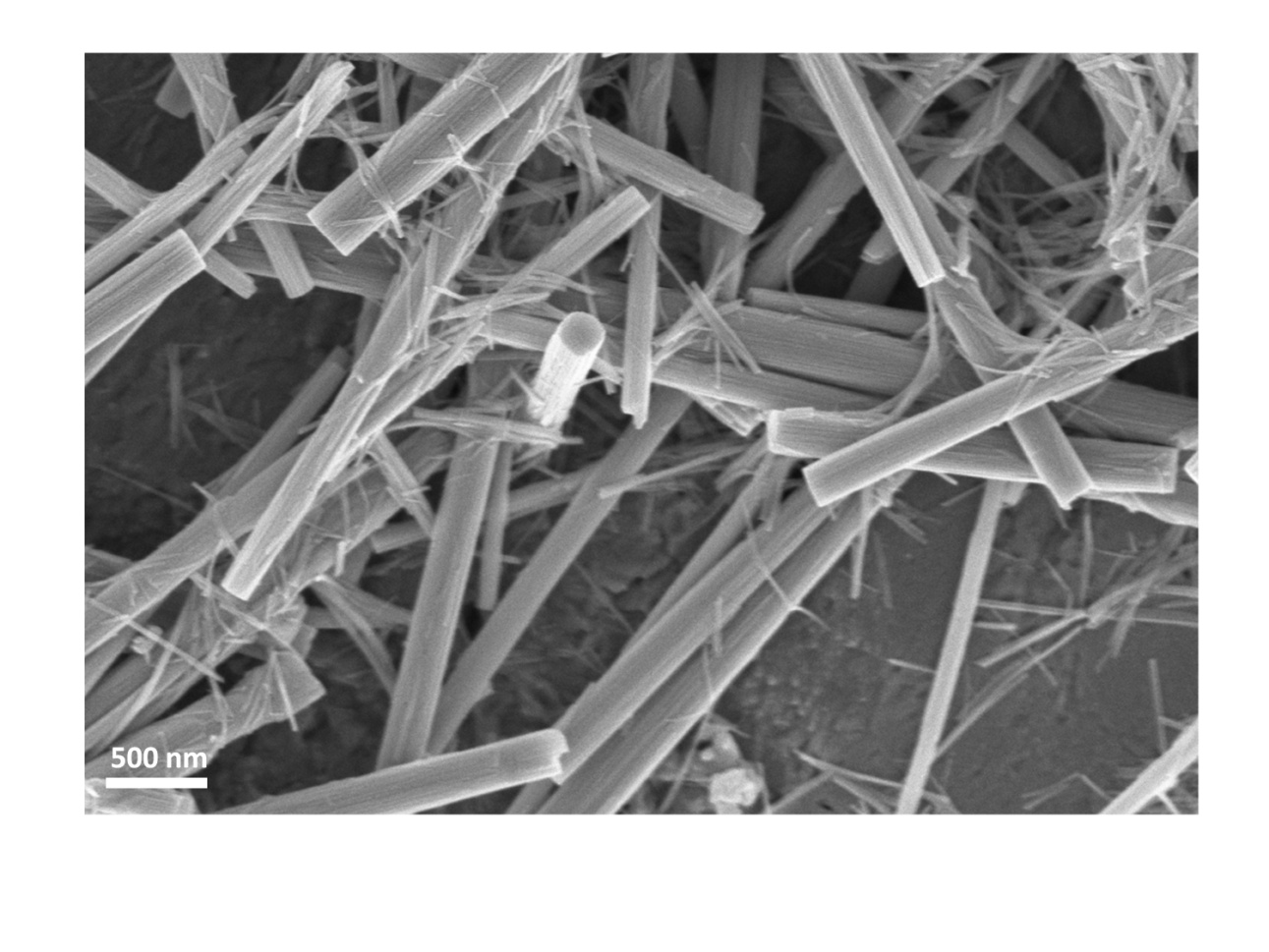


**Figure S2:** SEM image WO_3-x_ nanorods.


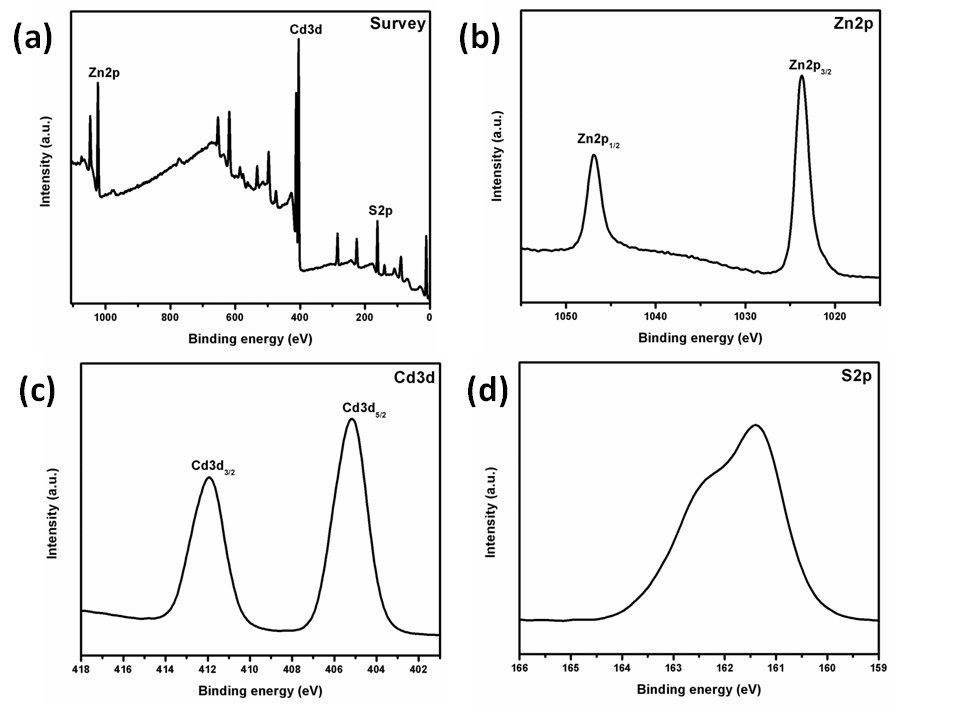


**Figure S3:** XPS spectra of Zn_0.3_Cd_0.7_S: Survey spectrum (a), Zn2p orbital (b), Cd 3d orbital (c) and S2p orbital (d).


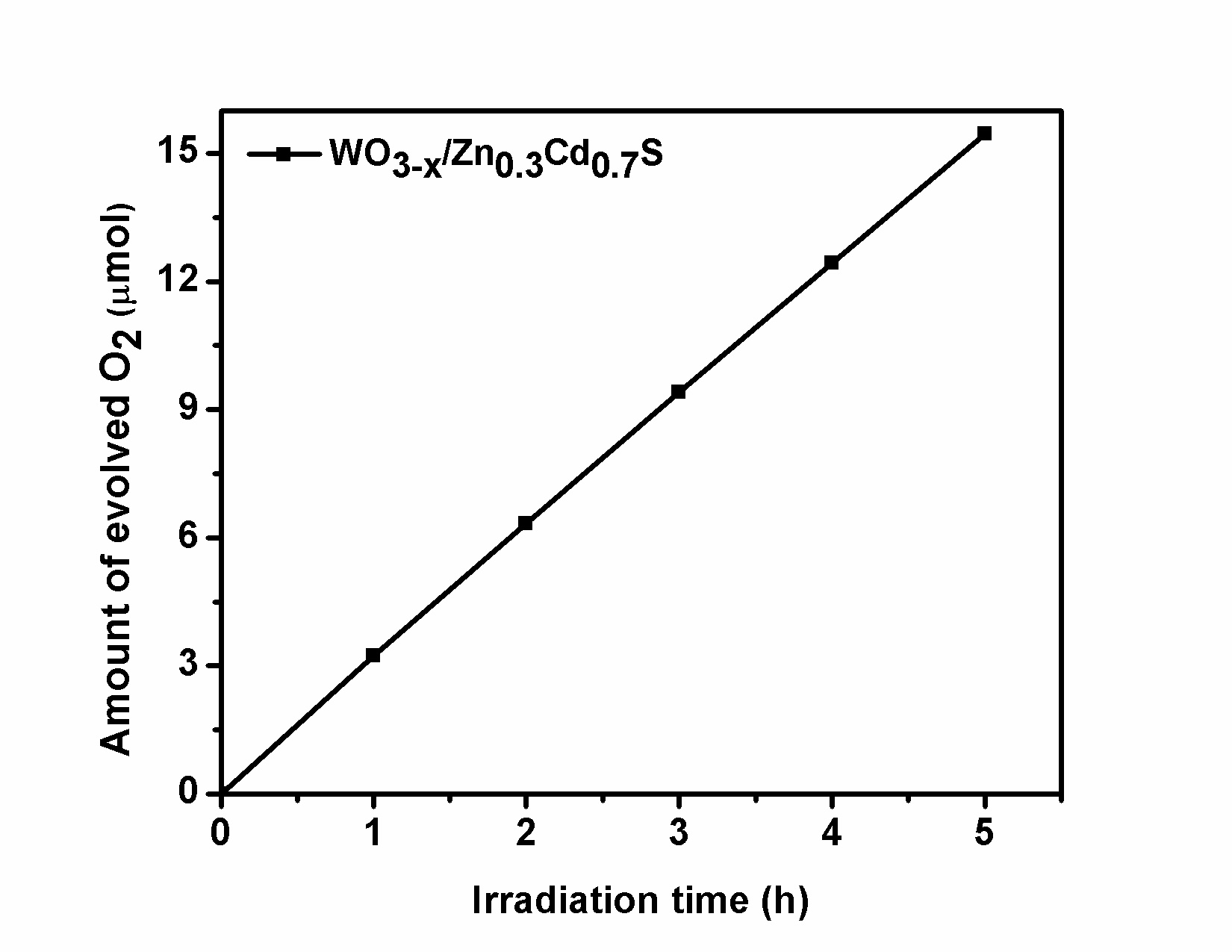


**Figure S4:** Time courses of photocatalytic O_2_ production from KIO_3_ solution on

WO_3-x_/Zn_0.3_Cd_0.7_S heterostructure.
